# Special Issue: “Microorganisms in the Environment”

**DOI:** 10.3390/ijms27052235

**Published:** 2026-02-27

**Authors:** Diby Paul, Udai Bhan Singh

**Affiliations:** 1 College of Life and Health Sciences, Truett McConnell University, 100 Alumni Drive, Cleveland, GA 30528, USA; 2 ICAR-National Bureau of Agriculturally Important Microorganisms, Kushmaur, Maunath Bhanjan 275103, Uttar Pradesh, India

Microorganisms are the unseen engineers of our environment. They regulate nutrient cycling, shape ecosystem structure, and mediate how natural systems respond to human-driven pressures. Over the past several years, it has become increasingly clear that microbial diversity is closely linked to key biogeochemical processes, including carbon and phosphorus cycling across terrestrial and aquatic ecosystems [1,2,3]. At the same time, advances in molecular biology, next-generation sequencing, metagenomics, and proteomics have reshaped environmental microbiology, allowing researchers to move beyond cataloging organisms and toward understanding the activities of microbial communities and their responses to disturbances [4].

Despite this progress, important questions remain unresolved. In particular, the long-term ecological consequences of agricultural intensification, chemical inputs, and land-use change are still difficult to predict, especially under field conditions and over extended timescales [1,4]. Similarly, although microorganisms are increasingly viewed as promising tools for remediation and sustainable resource management, their stability, adaptability, and effectiveness in complex environments remain incompletely understood.

Against this backdrop, this Special Issue of the *International Journal of Molecular Sciences* brings together seven contributions that reflect the breadth and direction of current research in environmental microbiology. Rather than addressing isolated problems, these studies engage with broader questions concerning anthropogenic pressures on microbial communities, links between environmental microbes and public health, and the practical use of microbial systems in remediation and biotechnology.

Several contributions examine how human activities and agricultural practices shape microbial communities. From our perspective, these studies are particularly timely, as they align with a growing recognition that management decisions can have lasting consequences for soil microbial structure and function [2,4]. Contribution 2 shows that herbicide application can substantially alter soil bacterial composition, affecting key functional groups. Contribution 3 further demonstrates that integrated rice–crayfish farming systems influence paddy soil microbiomes in a density-dependent manner, with clear implications for nutrient cycling and soil performance. Together, these findings underscore how routine management choices can reverberate through microbial-driven ecosystem processes [1].

The ecological and public health relevance of environmental microorganisms is highlighted in Contribution 1, which examines the occurrence of zoonotic microsporidian genotypes in water bodies associated with livestock farming. What stands out here is the role of the environment as an active interface between animal and human health. Recent One Health research has emphasized that environmental microbiomes can serve as reservoirs and transmission pathways for pathogens and antimicrobial resistance, particularly in agricultural and peri-urban landscapes [5,6]. Studies such as this reinforce the need to integrate environmental monitoring into broader public health and risk assessment frameworks.

Other contributions focus on the applied potential of microorganisms for environmental remediation. These studies resonate with recent work showing that native or well-adapted microbial communities often outperform single-strain approaches when dealing with complex contamination scenarios [2]. Contribution 4 illustrates the effectiveness of autochthonous microbial consortia in degrading polycyclic aromatic hydrocarbons in soil, highlighting the importance of community interactions. Contribution 7 complements this perspective by demonstrating how endophytic bacteria can be paired with plants to enhance phytoremediation, offering dual benefits for pollution mitigation and ecosystem recovery.

Beyond remediation, microbial communities underpin nutrient turnover, buffer environmental stress, and contribute to ecosystem stability, often responding rapidly to disturbance. Contribution 5 provides a synthesis of these services, framing microbes as central players in ecosystem resilience. At a finer mechanistic scale, Contribution 6 offers proteomic insights into anaerobic lignocellulosic biomass conversion, illustrating how microbial metabolic flexibility can be leveraged for bioenergy production and waste valorization [3].

We hope this collection will stimulate further interdisciplinary research and foster collaborations aimed at harnessing microbial processes for environmental sustainability. An important takeaway from these seven contributions is the direction they collectively suggest for the field: future research will increasingly need to integrate molecular-level insight with ecological context, embrace systems-based approaches, and translate microbial knowledge into solutions that are both scalable and environmentally relevant.
